# Exploring Australian pharmacists’ experiences with the electronic National Residential Medication Chart: a qualitative descriptive study

**DOI:** 10.1007/s11096-025-01894-3

**Published:** 2025-03-14

**Authors:** Jonathan Tan, Jasmine Tan, Leanna de Souza, Qianying Wang, Alexander Wong, Jarrod Mcmaugh, Kenneth Lee, Amy Page

**Affiliations:** 1 School of Allied Health, The University of Western Australia, Perth, Australia; 2 Business Development/Product Management, Strongroom AI, Melbourne, VIC Australia; 3 Pharmaceutical Society of Australia, Victorian Office, Parkville, VIC Australia

**Keywords:** Aged care, Digital health, Electronic medication charts, Health technology, Pharmacists, Qualitative

## Abstract

**Background:**

In Australia, the electronic National Residential Medication Chart (eNRMC) aims to enhance medication safety, reduce administrative burden, and communication in aged care facilities. However, research on its implementation is limited, with minimal pharmacist involvement, despite their critical role in medication management. It is essential to address the underrepresentation to optimise their experience with the eNRMC.

**Aim:**

This study aimed to explore the experiences of pharmacists who have used the (eNRMC) while providing care to residential aged care facilities.

**Method:**

A qualitative descriptive study design was employed, with thematic analysis conducted using The Framework Method. Pharmacists with experience using the eNRMC were recruited through purposive and snowball sampling and were invited to participate in semi-structured individual interviews in August 2024. Interviews were audio-visually recorded, transcribed using clean verbatim and analysed with NVivo software. An inductive coding approach was used to generate themes.

**Results:**

Twelve participants across Australia with varying levels of experience consented and completed semi-structured interviews. Three main themes were identified: (1) Improvements in Medication Management, (2) Limitations of the eNRMC Software, and (3) the Facility’s Transition to eNRMC Software. Pharmacists reported enhanced medication safety, workflow, and communication. However, eNRMC incompatibilities, restricted editing and increased workloads were challenges during implementation. Additionally inadequate training and resistance from general practitioners contributed to charting errors and medication incidents.

**Conclusion:**

Pharmacists from various roles described the eNRMC as beneficial for enhancing medication management, but software limitations and lack of support remain barriers that increased frustrations amongst users and impede adoption.

**Supplementary Information:**

The online version contains supplementary material available at 10.1007/s11096-025-01894-3.

## Impact statements

- This study helps to evaluate the benefits and limitations of the electronic National Residential Medication chart in practice.
- The study highlights the crucial role of pharmacists in medication management in aged care and how addressing usability issues could enhance the pharmacists’ ability to optimise patient outcomes.
- Understanding these challenges will help policymakers and healthcare leaders develop strategies to overcome these barriers to ensure that electronic systems become a seamless part of medication management.

## Introduction

Electronic medication management systems have been rapidly adopted internationally, replacing paper-based systems in clinical settings such as hospitals and residential aged care facilities (RACFs). In the US, 84% of long-term care facilities used an electronic system to record medication administration in 2019 [1]. In Macao, China, RACF staff reported fewer errors, simplified processes, and quicker communication after implementing an electronic medication administration system [2]. In Australia, most studies on the impact of these systems have been conducted in hospitals [3, 4], where they have been shown to improve prescribing practices by reducing errors and promoting more comprehensive prescriptions [5, 6].

The National Residential Medication Chart (NRMC) is a standardised chart used in RACFs across Australia [7]. In 2021, the Royal Commission into Aged Care Quality and Safety reported its recommendations to address the substandard care and systemic flaws identified in the Australian aged care system [8], prompting the national rollout of the eNRMC. Government grants to fund the transition from the NRMC to eNRMC began in July 2022 [9], with the aim to promote safe and accountable medication management by replacing paper charts and prescriptions with a fully electronic system [10].

The eNRMC enables electronic prescribing, and some systems also supporting medication dispensing and administration from a single document. Cloud-based eNRMCs also offer remote, real-time access to prescribers, pharmacists, and aged care staff. It can minimise errors by serving as a “single source of truth”, by allowing all healthcare professionals to access the most up-to-date and therefore accurate patient information instantaneously [10]. Other potential benefits include reducing administrative burden, facilitating interprofessional collaboration and using alerts to avoid potential medicine errors and send important reminders [1, 10, 11].

Currently, research evaluating the practical impact of the eNRMC in Australia is limited. Evaluating the eNRMC is crucial because older people are particularly susceptible to polypharmacy due to the prevalence of multimorbidity [12]. An umbrella review with data from six continents identified the global prevalence of polypharmacy was 45% in older people over 65 years [13]. Polypharmacy increases the risk of medication-related problems such as drug interactions, inappropriate doses or adverse drug reactions, which are often amplified in older people [14, 15].

Pharmacists play a critical role in managing residents' medications, from supplying medications to conducting Residential Medication Management Reviews (RMMRs) for aged care facilities [16]. This can result in reducing polypharmacy through describing [17, 18], which can lower the risk of complications such as falls [19]

The recent introduction and increased interest of the aged care on-site pharmacist role in Australia further signifies the importance of pharmacists in a setting where residents are at higher risk of medication-related harm [15, 16, 20]. However, current literature has captured only small sample sizes of pharmacists’ opinions of using electronic systems in RACFs [11, 21, 22]. Thisleaves a significant gap in research evaluating pharmacists' perspectives in using the eNRMC for medication management across diverse roles. A deeper understanding of pharmacists' experiences can enhance medication safety, workflow efficiency, and collaboration in RACFs, while also allowing system improvements, and targeted training for better implementation.

### Aim

This study aimed to explore the experiences of pharmacists who have used the eNRMC while providing care to RACFs.

### Ethics approval

This study was approved by the University of Western Australia Human Research Ethics Committee (2023/ET000003).

## Method

### Study design

To explore the perspectives and experiences of Australian pharmacists who have used the eNRMC, a qualitative descriptive study design was employed [23, 24]. This study is reported according to The Consolidated Criteria for Reporting of Qualitative Studies (COREQ) [25].

### Participant selection

Purposive sampling was used to recruit pharmacists practising in any role within Australia [26]. Snowball sampling was also used to reach a larger number and a wider variety of potential participants. Participants were recruited through direct contact with individuals (email) and RACFs (email and cold calling), as well as advertisement via social media (LinkedIn and Facebook). To enhance diversity in roles and demography, RACFs across the country were contacted, including RACFs in rural and urban settings, Participants and groups were contacted up to three times, with a one-week interval between each contact, before being deemed non-participants, as employing multiple strategies and repeated contacts has been shown to improve study participation [27].

A systematic review suggested that 9–17 interviews are typically sufficient to achieve data saturation in qualitative research [28]. Therefore, we initially aimed to recruit 17 participants. If all research team members mutually agreed that no new codes were emerging, which indicated data saturation, we would stop recruitment attempts early.

### Data collection and analysis

An interview guide was developed to explore the positive and negative experiences and potential areas for improvement (Supplementary File 1). Demographic questions were asked initially to provide the necessary context for the subsequent questions, which assessed their perceptions of the eNRMC. Data collection was commenced and completed in August 2024.

Prior to the start of the interview, all questions regarding the study were addressed. Written informed consent was obtained before each interview, and verbal consent was recorded at the start of each interview Due to the exploratory nature of this study, 20–50-min online semi-structured interviews were conducted to encourage participants to express their experiences. All interviews were conducted by JJ, a female Master of Pharmacy Student at UWA, with assistance from LD, a fellow female UWA Master of Pharmacy Student. In each interview, LD assisted with field notes and technical support. Neither researcher had any relationship with the participants.

Interviews were conducted over Microsoft Teams, using its built-in features for audiovisual recording and transcription. The interviews were conducted solely with JJ, LD, and the participant present. All transcripts were reviewed for accuracy by comparing them to the recordings, transcribed using clean verbatim and de-identified. Transcripts were not returned to participants for corrections. Repeat interviews were considered if requested.

Demographic information was collected from each interview, including location of practice, duration of eNRMC use, and role.

Two Master of Pharmacy students JT (male) and QW (female) conducted thematic analysis following The Framework Method [29], using NVivo software version 12 (QSR International Pty Ltd., Doncaster, Victoria, Australia). Transcripts were coded inductively, and codes were grouped to generate themes. Data were charted into a matrix, with each row representing a participant, each column representing a code, and each cell containing a relevant quote from that participant. This matrix was then used to draw connections and hence facilitate data interpretation.

### Ethical considerations

This study’s data were securely stored on a password-protected drive at the University of Western Australia, with access restricted to researchers named in the ethics application. To protect anonymity, all data were de-identified, and no identifiable details included.

To enhance the quality of data collection, JJ participated in interview training and practised while pilot-testing the interview guide with other team members. The pilot test was recorded and reviewed by a senior researcher who provided feedback on interviewing technique.

To enhance credibility during thematic analysis, analyst triangulation was employed, whereby both analysts independently coded each transcript before comparing their findings. Discrepancies in coding were discussed to achieve consensus. Changes to codes prompted a review of previous transcripts to ensure the inclusion of all relevant data and consistent application of codes. This process was repeated until no further changes were required. Peer debriefing with other Master of Pharmacy students, pharmacy academics and clinicians external to the research team further enhanced credibility. A codebook was used throughout coding to minimise coding drift between and within researchers, hence improving confirmability. An audit trail was maintained to ensure dependability.

## Results

### Participants

A total of 17 pharmacists expressed interest in participating in the study. Out of the 17, 12 pharmacists provided written and verbal consent for interviews to be conducted. Reasons for non-participation included lack of remuneration (1 participant), ineligibility due to their role (1 participant) and lack of time (3 participants). Data saturation was achieved by the 10th interview and a further two interviews were conducted to confirm saturation. Participants were classified into three groups based on their roles, as shown in Table 1. Participants’ demographic information is presented in Table 2. Interviews ranged from 21 to 48 min in length and had a mean duration of 36 min, and no repeat interviews were conducted.

**Table 1:** Types of pharmacists and their roles within a RACF

Types of pharmacists	Role
Residential Medication Management Review (RMMR)	Accredited Pharmacist that identifies, resolves and prevents medication related issues (24)
Supply Pharmacists	Pharmacists that dispense and provide medications for RACFs
Consultant Pharmacists contracted with RACF organisation	Pharmacists that are contracted to provide advice to a RACF organisation as a whole

**Table 2:** Demographic information of the participants. N = 12

Demographics	n
*Sex*	
Male	3
Female	9
*Type of pharmacist*	
Residential Medication Management Review	4
Supply Pharmacist	6
Consultant pharmacist contracted with RACFs	2
*Years of experience with the eNRMC*	
0–5 months	3
6–11 months	2
1–2 years	3
2 + years	4
*State of Practice*	
New South Wales	1
Queensland	2
South Australia	3
Western Australia	3
Victoria	2
Unknown	1

### Themes

**Fig. 1:**
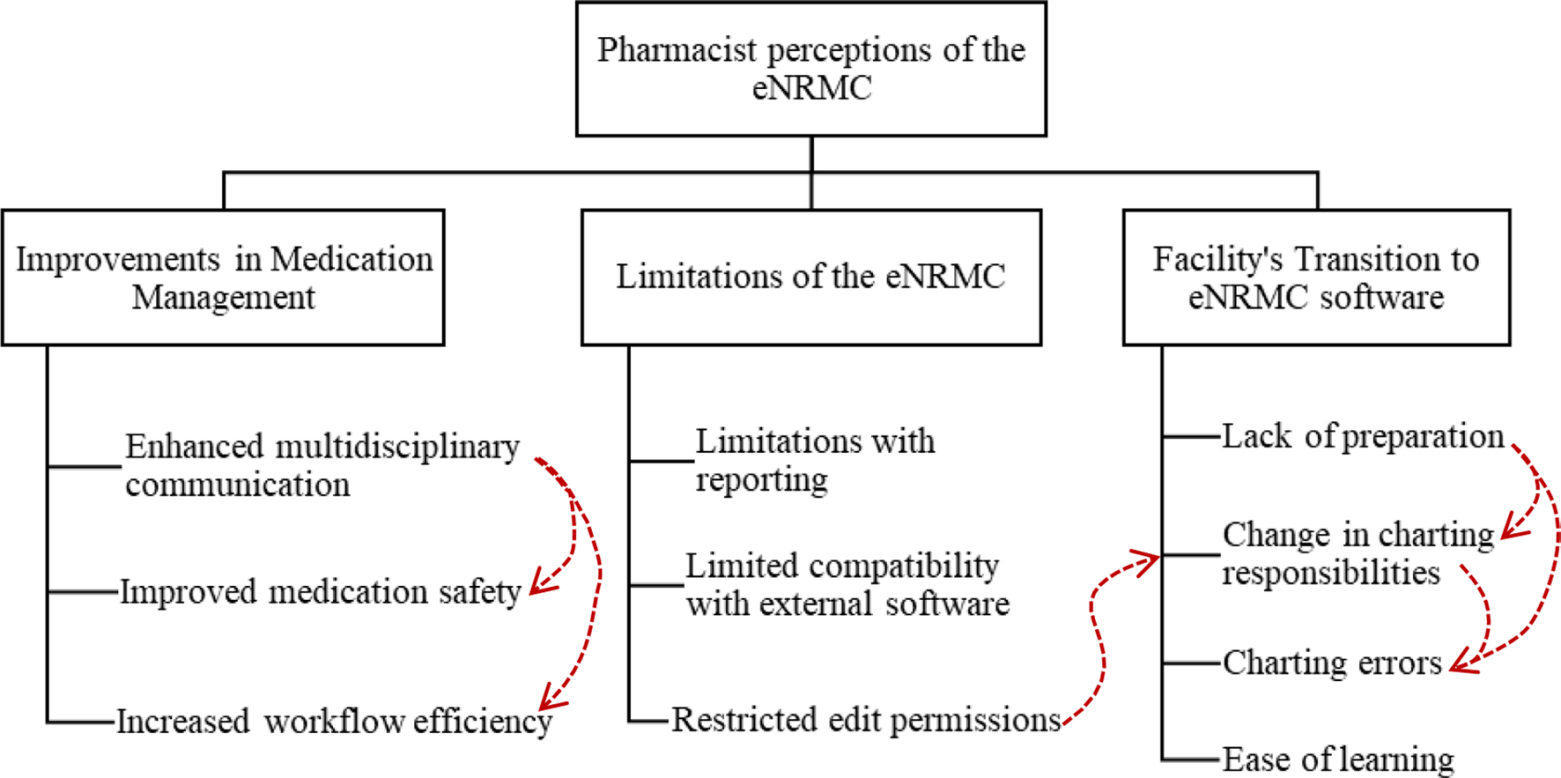
Themes and Subthemes identified from interviews. Red dotted lines are used to indicate how one subtheme is related to another subtheme

Three themes were identified after the analysis of the interview transcripts: Improvements in Medication Management, Limitations of eNRMC Software and the Facility’s Transition to eNRMC Software (Fig. 1).

### Improvements in medication management

#### Enhanced multidisciplinary communication

Before the eNRMC, pharmacists were required to contact the RACF and for messages to be passed onto the relevant person. The eNRMC provided different avenues that enabled quick communication with other users of the system. This included comment boxes and direct messaging capabilities. With the eNRMC, pharmacists were able to contact the relevant person directly, which allowed for efficient communication.

“*[For paper charts] I would ring the nurse and …I'm completely reliant on the nursing staff to make that note and to then have that conversation with the doctor and action it…but using the eNRMC…I can write a note on the patient's profile to the GP” -P 12*.

However, communication capabilities were dependent on the type of eNRMC software.

“*In [ProgramX], if a doctor has prescribed something electronically…but not put a strength on it. What the pharmacist can do is…send a little note through to the doctor to say, “please confirm that this is your intention”…[Yet for ProgramY and ProgramZ], we still need to call or e-mail the doctor if there's issues.” - P2*.

#### Improved medication safety

The eNRMC’s formatting clarified prescribers’ intentions as it encouraged prescribing in a more complete manner. This included features that forced prescribers to input indications and start and end dates for short course medications. Being electronic, the eNRMC also enhanced chart readability.

“*The electronic chart has some availabilities for the GPs to actually write indications… The electronic chart has allowed a bit more clarity around indications for PRNs [pro re nata or when required] definitely…. Sometimes when you get the handwritten charts, they are illegible. They don't specify the form of the medication, whether it's immediate release or the modified release”* -P1.

Pharmacists were also able to benefit from medication warnings for special handling, special instructions or allergies. Alerts were also available for notification of chart changes and timely dose administration.

“*If a resident has to have all their medicines crushed, sometimes the chart gives clues like in red with their star visually means that you can't crush it. Cytotoxic medicines, they come up in purple, so you know to do that as unpacked straight away*”-P10.

#### Increased workflow efficiency

One of the key capabilities that was complimented on was the ability for changes to be almost instantly updated for all users. All pharmacists used web-based systems, which allowed access to the eNRMC from locations away from the pharmacy or RACF, whilst also having the most up-to-date chart information. The pharmacists also mentioned the doctors, who were able to chart and make changes away from the RACF.

*“I'd be off site potentially in a [multi-disciplinary] meeting… I could log into… the electronic chart and check in real time while we're in meetings”* -P11

Supply pharmacists were able to transcribe off the eNRMC to dispense medications. The increased accessibility of the eNRMC for doctors allowed for medications to be dispensed in a timely manner whilst not being hindered by handwriting readability.

“*If someone's being put on antibiotics at 5 o'clock on a Friday afternoon, we don't have to worry about a script…the minute the change occurs, we just get it done and we send it up. We don't have to wait for any paperwork.*” -P8.

The ability to transcribe off the eNRMC and to be able to always have a prescription reduced the need for administrative communications, such as requesting, faxing and emailing prescriptions or following up on stock orders. Nurses were also able to automatically submit medication orders on the eNRMC and track order statuses, which further reduced stock management communications.

“*As the GPS does a change, we can immediately see it, especially for urgent medicines…There's no calling the doctor “When's the script coming through?*”-P5

“*The biggest benefit is it’s cutting out the nurses in terms of them having to do that admin step…having to fax the charts and so that's reduced their burden…[The eNRMC] has a resupply function.*” -P12.

### Limitations of software

#### Limitations with reporting

A high-quality history is required for audits to be worthwhile. The eNRMC provided automatic auditing functions, however, a few pharmacists found that their system’s history was quite limited or missing. Another issue faced was when medication courses ended or medications were not renewed, it became difficult to gather information about that medication’s usage.

“*I can't actually quickly see what are all the changes over the last six months….I've gone into nursing homes and talked about the medicines and then they go “oh, yes, this was stopped last week”…[But] I didn't pick that up from the electronic chart.*” -P1.

#### Limited compatibility with external software

Although the eNRMC allowed for medications to be readily dispensed, some pharmacists were disappointed that the eNRMC was not compatible with their dispensing software or packing software. Pharmacists expected auto-transcription as a feature to reduce errors.

*“You're transcribing onto packing software, so that lack of integration from the electronic charts is a challenge…it's disappointing that we can't do better with the integration of those systems”* -P11.

A few of the pharmacists had issues using a hybrid system, where progress notes, the eNRMC and medication administration were separate electronic systems and were not linked to each other. For this system, the supply pharmacists must dispense from the eNRMC and then ensure changes were made on the administration system. This resulted in duplication of information, charts not being filled in and updates not being uploaded.

“*[The doctor] writes their electronic script for some Trimethoprim on a Saturday night, the pharmacy doesn't get that notification until they're in next…. it didn't end up going on to the electronic [administration] chart…the person ended up not receiving their antibiotic and they ended up in hospital with quite severe cystitis.”*-P1.

#### Restricted edit permissions

Most pharmacists have voiced their complaints of not being able to make changes on the eNRMC. The only user that has permission to make changes are the GPs. This increased the reliance of the prescribers to maintain the chart. GPs were also expected to edit the chart for external doctors, such as after-hours doctors or specialists, as they don’t have access to the eNRMC.

*“We have to annoy the doctor to get that changed on the chart itself so that the nurses can administer it when they do their check offs…[ProgramY] does not have the function for us to switch the form”* -P8.

To help alleviate the reliance on GPs, some pharmacists wanted to be involved and were hopeful for a drafting system to be able to provide recommendations.

“*I think opening access to the supply pharmacist being able to access the charts would make a huge improvement… if they have received a prescription from a hospital or an external provider like a locum or a dermatologist, if they can chart that medication on behalf of those GPs or prescribers.*” -P6.

### Facility’s transition to eNRMC software

#### Lack of preparation

Implementation of a new system was described as large workload for all healthcare professionals. The pharmacists that were directly involved with implementation and transition of the eNRMC all agreed that transcribing was one of the largest tasks. The high workloads led to frustrations, especially amongst the GPs. This led to GPs not engaging with the training material, which have also been regarded as inadequate by GPs and pharmacists. Pharmacists reported that GPs either did not have time to do the training or were not fully committed to engaging with the material.

“*We received a module to complete….it was a 6 h [online] module so we all had to complete that before we went live…the links were sent out to the GPs, but the GPs don't have 6 h to do the training… so they went in quite blind.”* -P6.

“*No one was educated in how to chart on the eNRMC properly. We had a lot of meetings with the doctors and… the software company multiple times…they send in instruction videos, but they're pathetic.”* -P3.

However, before they could use the eNRMC, pharmacists and GPs reported the difficulties of gaining access to the eNRMC for the first time. This led to further frustrations and delays in implementation.

“*A whole heap of different criteria just to log in the first time and they weren't able to. And that got them frustrated and offside straight away.”* -P4.

#### Change in charting responsibilities

As previously mentioned, the workload for GPs increased during the transition stages and post-implementation of the eNRMC. Due to restricted edit permissions, GPs were expected to have full responsibility of the eNRMC as they became the only healthcare professional that could edit the charts. This led to GPs voicing their frustrations as they did not want sole responsibility of the chart, and some became resistant to the changes. These GPs refused to use the eNRMC to its fullest capacity and a minority retiring to avoid learning and working with the new system.

“*Some of them who were contemplating retirement…. “This is too much for me. It's time for me to hang up my shoes”… I'd say probably 95% of the doctors have come on board and they're happy to use it”* -P2.

#### Charting errors

With the reported lack of adequate education, low engagement with education material, and GPs being resistant, many pharmacists found an increased incidence of charting errors especially at the start of using the eNRMC. This included dosing, medication timing and chart duplication errors. However, a lot of these errors have been reported to be transient until all users become more accustomed and experienced with the eNRMC.

“*The biggest difficulty we've had is the GP barrier and them not wanting to use it and it has probably led to at least five direct medication incidents, including incorrect doses given, extra chemical restraints given…someone was given a restrictive practice for longer than they were supposed to…a blood thinner that wasn't increased when we thought it had been increased.”* -P6.

#### Ease of learning

Although there were many issues with the education behind the eNRMC, many pharmacists found that the system was intuitive, simple to learn and that users simply required time to get accustomed.

“*Even the most technologically challenged person can get the hang of it. We've got some older GPs who've just embraced it. Some older RNs who might not even be competent with an iPhone but they're getting the hang of it and doing really well.”* -P7.

## Discussion

### Statement of key findings

This study explored pharmacists' perceptions of the electronic National Residential Medication Chart (eNRMC) in residential aged care facilities. Our findings were categorised into three overarching themes: (1) Improvements in Medication Management, (2) Limitations of the eNRMC Software, and (3) The Facility’s Transition to eNRMC Software. Each theme encompassed various sub-themes that provided deeper insights into pharmacists' experiences with the eNRMC.

### Interpretation

Pharmacists reported enhanced medication safety and improved workflow efficiency through the eNRMC’s capabilities, which thus allowed prompt response to medication changes and optimised the patient care process. Such capabilities included improved readability, automatic alerts, real-time updates and reduced administrative communications. These findings are consistent with existing literature, highlighting the benefits of electronic prescribing and medication chart systems [11, 30]. Similarly, a study conducted in Macao, China, reported that implementation of an electronic medication administration record system enhanced patient safety by reducing errors in prescribing, dispensing, and administration [2]. Our study demonstrated that the speculated benefits of the eNRMC have been achieved in practice, as shown by the findings.

Despite the potential enhancement to medication management, pharmacists have identified several limitations that hindered the eNRMC’s full potential. Reporting limitations and integration challenges disrupted the flow of information between different healthcare providers, which resulted in fragmented care and increased administrative burden. This aligns with a previous study investigating the adverse impacts associated with the introduction of electronic health records, where similar concerns about technical issues were raised [21]. The restricted access for pharmacists to make modifications on the eNRMC significantly limited their ability to contribute their specialised knowledge in medication management. Especially in older people, who require more monitoring and care, this constraint diminished the pharmacist’s role in ensuring medication safety and optimising pharmacotherapy. Furthermore, instead of streamlining charting, this limitation shifted a greater administrative burden onto GPs, who now must solely make all necessary medication adjustments. This compounded by the lack of adequate training increased frustrations resulted in the observed resistance against the system’s adoption. Consistent with previous literature, the low uptake by GPs due to increased documentation burden and inadequate training can influence other staff members’ attitudes towards the system, creating a ripple effect that further impedes successful adoption [11, 21]. These factors collectively hinder the successful adoption of the eNRMC, interdisciplinary collaborations and reduces the overall effectiveness in medication management. Addressing these access limitations, with the appropriate regulatory parameters, could enhance workflow efficiency, reduce prescribing errors, and ultimately improve patient outcomes.

### Further research

Our study contributes to existing literature by providing empirical evidence of the practical advantages and unforeseen limitations of the eNRMC in real-world settings. These findings have important implications for the future of medication management in RACFs. The eNRMC has shown potential for widespread adoption by improving medication safety and workflow efficiency. However, limitations such as lack of integration, increased documentation burden and restricted user access hinder implementation. Future research should focus on addressing these challenges to enable smooth integration of the eNRMC in RACFs. Insights from pharmacists’ experiences can guide policymakers and healthcare providers in optimising medication management practices and improving care quality in aged care settings.

### Strengths and limitations

Despite attempts to recruit a variety of pharmacists, akey limitation is that our data may not be representative of the views of pharmacists across Australia, as we lacked participants from Tasmania, Northern Territory and Australian Capital Territory. Furthermorenone of the participants were in an aged care on-site pharmacist position, a role recently introduced in Australia. Future studies could explore aged care on-site pharmacists’ perspectives of the eNRMC, as their clinical experiences may differ from those of community pharmacists, who often highlight logistical aspects of the eNRMC [15, 16, 20].

As our findings are based on self-reported data, social desirability bias may have led participants to overreport positive aspects or underreport the challenges experienced of the eNRMC. These findings would not adequately represent the pharmacists’ actual needs and would limit the effectiveness of interventions. To mitigate this, interviews were conducted by a researcher unknown to the participants, with confidentiality procedures explained to concerned participants. Although the interviewer lacked research experience, she underwent interview training from a supervisor with extensive research experience and discussed each interview with the research team.

A qualitative inductive descriptive study design minimises researcher bias by aligning closely with the data. To address the potential coding drift risk inherent to inductive study designs, we consistently referred to the research question and used a codebook. We also employed analyst triangulation, maintained a single codebook with an audit trail, and conducted peer debriefing to ensure the rigour of data analysis [20, 31].

## Conclusion

While many pharmacists appreciated the potential for improved medication safety and workflow efficiency, concerns regarding lack of training and software limitations remain barriers to widespread acceptance of the eNRMC. Facilities should acknowledge that transitioning to the eNRMC is complex and should be fully implemented only when staff are confident and competent with the new processes. Acknowledging the value of pharmacists in aged care and addressing these challenges by providing targeted support and resources for pharmacists, who provide specialised expertise in medication, can improve patient outcomes. Additionally, ongoing evaluation of eNRMC implementation can help identify best practices and areas for improvement, ensuring that the system continues to evolve in response to the needs of pharmacists and aged care facilities.

## Supplementary Information

Below is the link to the electronic supplementary material.

Supplementary file1 (PDF 315 KB)
