# Supplementary material for: Exploring Australian pharmacists’ experiences with the electronic National Residential Medication Chart: a qualitative descriptive study
Source: Int J Clin Pharm. 2025 Mar 14;47(5):1195–203. doi: 10.1007/s11096-025-01894-3 (PMC12432027; doi:10.1007/s11096-025-01894-3)
Supplement: Supplementary file 1 — Supplementary file1 (PDF 315 KB) [file 11096_2025_1894_MOESM1_ESM.pdf]

Interview guide.

- Can you tell me about where you were working when you used the eNRMC?
    - Are you contracted to a residential aged care facility?
    - (If not, but they have encountered eNRMC): was it for a resident you service regularly or a one-off supply?
    - Have you used the eNRMC in any other setting?
    - Did working with it in different settings change your experience with it?
- How do you use the eNRMC in your daily practice?
- Can you tell me why your workplace implemented the eNRMC in the first place?
    - How long ago was it implemented in your workplace?
    - How has the introduction of the eNRMC changed the way you work?
    - (If they mention comparison to previous system): what system were you using before eNRMC?
- What positive experiences have you had with the eNRMC?
    - Are there any specific examples or scenarios?
    - Why was this beneficial or useful to you?
- Have you had any difficulties with using the eNRMC?
    - What caused these difficulties?
    - What was the impacts of these problems?
- How do you think the eNRMC could be improved?
    - Are there any features in particular which you would like to see implemented?
    - How would this be beneficial?
- Do you have any other feedback regarding the eNRMC?
